# Experiences of Being a Couple and Working in Shifts in the Mining Industry: Advances and Continuities

**DOI:** 10.3390/ijerph18042027

**Published:** 2021-02-19

**Authors:** Jimena Silva Segovia, Pablo Zuleta Pastor, Estefany Castillo Ravanal, Tarut Segovia-Chinga

**Affiliations:** 1 Escuela de Psicología, Facultad de Humanidades, Universidad Católica del Norte, Av. Angamos 0610, Antofagasta 0610, Chile; castilloravanale@gmail.com (E.C.R.); tarut.segovia@gmail.com (T.S.-C.); 2 Escuela de Psicología, Universidad Bernardo O’Higgins, Av. Viel, Santiago 1497, Chile; pablozuletapastor@gmail.com

**Keywords:** couple, shift work, gender, Chile

## Abstract

In this study, we sought to understand, from a gender perspective, the experiences of mining couples in Antofagasta, Chile, especially the balance between their intimate lives and the absences of their partners due to the shift work modality. We analyzed testimonies from men and women living in Antofagasta, which is considered to be one of the world’s three largest mining regions. Among the main findings, power relations based on the hegemonic gender model supported by the sexual division of labor were identified, which persist in this mining area, despite progress in gender equality issues in Chile. Although there are differences between the discourses of men and women and their subjective positioning, we propose that both actively collaborate with the reproduction of social gender relations marked by male domination. We propose that the way in which couples live is associated with the organization of mining work and especially the shift system, which is central to the reproduction of the gender order with a heteropatriarchal tone.

## 1. Introduction

**Figure 1:**
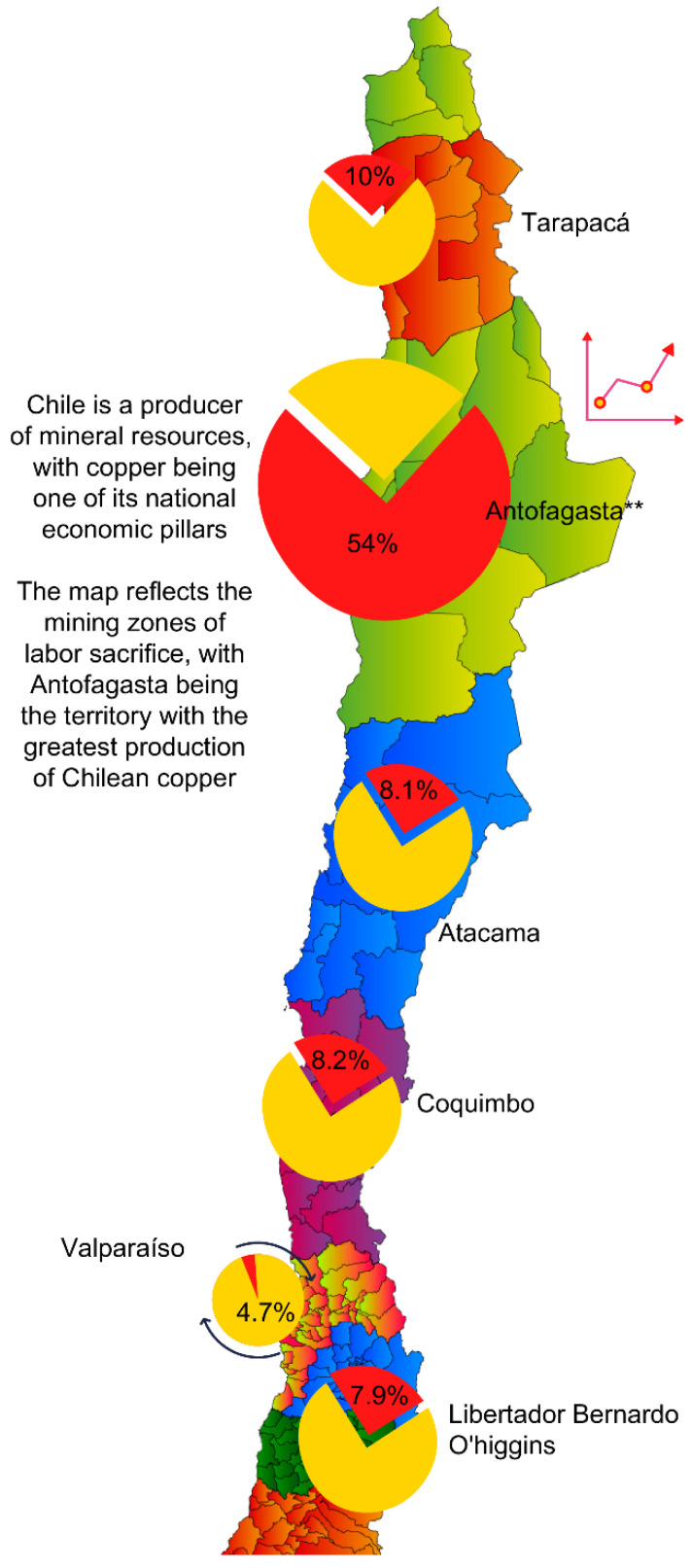
Map 1: Represents copper production in Chile. Source: Cochilco Ministry of Mining. Estrategic Investments 2020–2028.

The Chilean copper mining industry is deeply linked with the socioeconomic and cultural structures of territories where large-scale mineral extraction processes occur. The Antofagasta Region is located in the north of Chile and is one of the main copper mining areas in the country (see Figure 1) [1]; the location illustrated in map 1 represents the copper production in Chile. A significant number of families living there base their economic livelihood on the various sectors associated with mining, which ranks first in the generation of employment in the region at 31%, contributing to 57.9% of the regional Gross Domestic Product (GDP) [1,2]. The cities of Antofagasta and Calama stand out worldwide for their levels of copper production, ranking third among 68 ore-producing countries [3,4].

In the last 10 years, the region has become the second most important in Chile, with an economic growth rate of 13.1% [2]; however, these data are in contrast to the assessment that the inhabitants make, for example, of their security and human development. Regarding this, the Regional Observatory for Human Development has pointed out the presence of a general atmosphere of insecurity and the persistence of different social and economic gaps in education, access to work, housing, and health among the region’s inhabitants. Likewise, in a study on social perceptions, a lack of interest was observed in the inhabitants’ desire to take root in the locality. This process is associated with a high rate of floating population, i.e., those who work in Antofagasta, but their families live in other regions. These groups are attracted by the mining work and consider the region to be optimal for work but not suitable for family life. The floating population corresponds to 40% of the inhabitants [1,5].

When reviewing other research carried out in Chile on relational processes, we discovered that the findings reveal, on the one hand, a social network crossed by typical elements of the contemporary neoliberal model, where the value of money and consumption is placed above relational quality. On the other hand, gender relations are strongly marked by historical androcentrism. These cultural marks are expressed in the structure of the northern culture, which helps to reproduce gender and class hierarchies articulated with conservative values, thus stressing the emotional relationships between couples in the area. In the same context, progress can be observed in these restrictions toward equality in the youth population, which are framed by various expressions of violence [6,7,8]. This resistance to recognizing the capacities and rights of women is exemplified by the persistence of social representations generated in the mining environment, such as rejection, contempt, and an exclusionary perspective to their presence in these labor territories, with emphasis on operational areas or maintenance, considering that they are not “places for women”. In the past, their participation was limited to domestic functions, such as cleaning, food preparation, and administrative or secretarial work [9,10,11]. Although they have now entered into professional jobs, due to the pressure of changes in Chilean labor policies, their presence and the valuation of the female subject continue to be slow and conflictive, as can be seen in the experiences outlined in this study.

Likewise, affective relations are organized around the possibilities and limitations characteristic of the organization of mining work. Therefore, for the purposes of this study, we use the notion of “mining couples or families” [7,12,13,14]. Part of the slowness in the transformation toward equality in couples in the mining industry is related to economic–emotional dependencies linked to important social and wage benefits offered by mining companies. We also suggest that mining jobs involve labor relations that, along with demanding high levels of productivity from the workers, imply periods of exclusive dedication to the worksite when working shifts—shifts are organized in periods of days exclusively for work and the same number of days of rest, which vary according to the activities that the worker performs and the type and size of the mining company; they can also be 12 × 12, 7 × 7, or 4 × 4—far away from the cities where their families live [7,15,16].

We also confirm that in local social discourse, “mining couples” are signified as holders of high levels of economic resources, which associates mining workers with the role of providers and, quite frequently, the sacrifice of family and couple experiences. This way, the gain in economic resources has affective costs for the miner and his partner—a high cost in terms of the dynamics of absence/presence of the man at home, resulting in a strong contradiction: working for the family at the cost of the family. Research shows that this type of relationship can be associated with the libidinization of money within couples and families, converting money into an important means of communication among men, women, and children, and many times forming the ultimate basis for conserving the familiar bond [16].

In traditional mining couples, it is principally the men who are absent from family life due to shift work, during periods that range from four days on the worksite and three days off to working shifts of two uninterrupted weeks (biweekly period) (Labor Code, article No. 153, 2005). In this dynamic, in the absence of their partners, women concentrate power in the private sphere, related to decision-making in their day-to-day relations with their children, as well as in the administration of the economic resources that come from mining work; however, when their male partner returns from his shift, this meager quota of power for the woman is retracted, re-establishing hierarchical gender positions—a system that has been labeled the “accordion family” [14,17]. This denomination refers to the dislocation between work and time off (life as a couple and with the children) and leads workers to experience emotional distancing from the family [12]. The excessive physical demands historically associated with this activity, in addition to risks for health (and even life) and extreme environmental conditions (thermal oscillation, snow, and geographic altitude, among others), were thought of as constrictions that women could not endure. It is undeniable that shift work impacts the subjectivities of men and women, and certainly social/family and gender relations. In this dynamic, work shifts filter into the affective bonds, leaving marks that lead to the emergence of power conflicts in a couple’s relationship [6,12,18,19,20,21,22].

In this framework, various studies, in Chile and other countries, are emphatic in showing how the production of subjectivities and relations based on male domination [23] cannot be understood outside the socioeconomic, cultural and historical context in which the men are found [24], as well as the productive context. In this respect, the authors of [25], in a study on the production of masculinity in oil work in the Argentine Patagonia, give an account of how the work organization is not limited to the unrestricted extraction of natural resources or to the exploitation of the work force, but rather, at the same time, produces a form of manliness according to the company’s objectives; that is, male subjectivities intended to systematically increase productivity and the company’s profitability. In the words of Dejours: “the contemporary evolution of companies’ forms of work organization, management and direction is supported, after the neoliberal shift, on principles that precisely suggest sacrificing subjectivity in the name of profitability and competitiveness” [26]. Along similar lines, [27] shows how the company figure, in its desire to increase productivity and profitability, requires men to sustain their identity as being able to endure the working conditions through a systematic desensitization process described as virilization of the subjective body, leading to the reproduction of male domination and of gendered and hierarchized relations that transcend the boundaries of work, also marking and defining social and family relations in terms of more or less manifest conflicts of power that over and over again position women and other feminized subjects in positions of inferiority.

In addition, in 2015, Leiva and Comelin [28] gave account of how the shift system constitutes one of the main obstacles to making progress in the balance between work and family in mining contexts, showing how the company where they carried out their research stated that the measures suggested for progressing in the matter were impracticable. In this respect, the organization of the work and mining production is contributing to what [29] defined as hegemonic masculinity, in which the aspiration to be a “real man” implies subjectively positioning himself above others, whether women or other men. Paraphrasing a classic study by Gilmore [30], the mining production system and, especially, its shift system constitute tests of masculinity. They require male workers to be, and to demonstrate that they are, men, in both the public-working and private-domestic spheres, constituting the concept of a mining family, where the constitution of the family and its internal conflicts of power are not separate from the production system.

In Chile, such conflicts take on even greater relevance when we consider that in the world, but very specifically in this country, May 2018 was the scenario that marked the start of what has been called the rebellion against patriarchy [31], protagonized by women, which was a movement that consisted precisely of radically making an issue of the structure of patriarchal domination and its symbolic violence [32]. Considering this sociopolitical process and focusing on the perspective of this work, while strictly speaking, the qualitative research prescinds a hypothesis according to its epistemological base, our research purpose is based on the significant cultural shifts occurring in the country. These have been leading to more visible transformations in the androcentric conservatism of the mining family, especially in younger families who start to seek new adjustments and arrangements in their life as a couple. Our research question was thereby formulated as follows: Which cultural impositions of a traditional gender nature associated with the mining family are reproduced and which are transformed in the discourses of male mining workers and those of the women-partners?

## 2. Materials and Methods

Considering the research question, we point out two important methodological directions. First, we make discourse on mining work, its social and technical organization, and its implications for life as a couple the object of our study; second, apply the technical analysis of the discourse from a gender perspective as a technical tool [33,34,35,36].

### 2.1. Regarding the Information Production Strategy: The Interviews

We employed a biographic strategy [37,38] using an investigative-type account, emphasizing life as a couple and mining work. Each session lasted 120 min, with three encounters with each participant, regarding dimensions such as the demands of mining work, tensions between intimate life and work, and affective relations and resolution of conflicts. Each person signed an informed consent form that ensured the protection of their anonymity and authorized the use of their information for research purposes. Each recorded interview was transcribed and constituted analysis material for the preparation of the next session. We started the interviews with a word game technique for 20 min—a card with a motivating word. For example, we used the words: love, child, work, home, sex, couple, etc. The participants were asked for free association and, in that way, we opened a space of trust, reducing the tension, which enabled expanding to other, more complex topics in the couple’s relationship. This phase enabled going into the referenced dimensions in the remaining 100 min.

### 2.2. Regarding the Analysis Strategy: The Option for Critical Analysis of the Discourse

Since the research question refers us to the arrangements achieved in the intimate life of the couple, we analyzed what the discourse reproduced and/or transformed regarding gender order in the arrangements of life as a couple. The focus of this study was on the capacity that the discourse has to produce or reproduce gender power relations [39]. In this sense, when we focus the research question on the topic of how cultural discourses facilitate a reproduction and/or are transformed at the center of our research question, we would somehow understand gender as *interdiscursive*, spoken and being spoken through and by the discourse of the participants who express it. From this perspective, the speaker is conceived as a “subject inserted in a social topography that defines places of expression that are fundamentally positions of subjectivity” [40].

**Figure 2:**
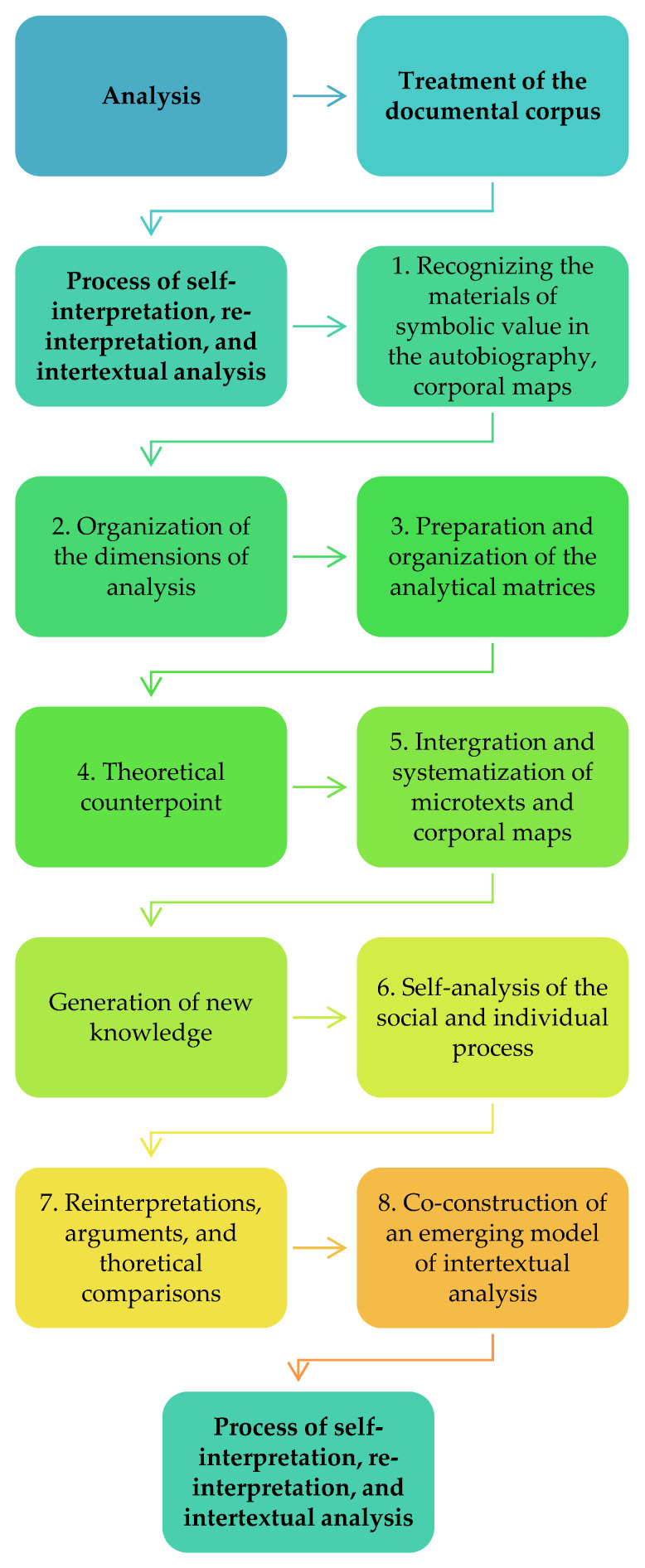
Analysis procedure phases. Source: self-prepared.

Consequently, we sought to understand such subjective positionings, which Davies and Harré [41] defined as conversational phenomena, as a social interaction capable of generating social results. Standing out among these results are interpersonal relationships, since the subjective positioning implies at the same time the positioning of other subjects and social objects. Consequently, through positioning, social orders are generated or regenerated. From this perspective, conversation is understood as a set of acts of speaking; that is, it involves expressions with illocutive strength, capable of producing social effects [42]. The social effects that interest us here reproduce the patriarchal domination inscribed in social gender relations and those that collaborate in their transformation. To analyze these processes, we have followed an order of 8 phases illustrated in the following diagram (Figure 2):

With the material from the different encounters, we started analyzing the discourse [39] identifying emerging focuses that were organized in matrices (see Table 1) for reordering in conflictive knots, gender tensions, linked emotions, search objects, and interpretations. This organization enabled advancing toward interpretation [35,36,37]. The matrix was fed with fragments of narratives for interpretation, those most significant for the problem being studied. In the process, we emphasized the search for emotions linked to the couple’s interactions.

### 2.3. Participants

During the 2018–2020 research process, 75 people participated in interviews and conversation groups, from which we have selected 11 between mining workers and women affectively linked to them, for the specificity of their narratives regarding the topic proposed for this article on advances and continuities in couple relations. The age distribution was from 21 to 65 years old, including persons in middle-management positions from the mining sector and in the lower-middle socioeconomic sector who agreed to be interviewed, given that they are the population with the biggest emotional, family, and couple voids, as opposed to persons at management or higher levels, who have access to greater cultural and symbolic capital [23], with more opportunities and flexibility in the labor sphere and therapeutic treatment, if applicable (see Table 2). The criteria for inclusion included being a mining worker or a mining worker’s partner, being subject to a shift system, being in an emotional relationship for more than one year and having their own children or their partner’s children.

We reached out to institutional networks related to mining companies and their unions to access the persons interviewed. We used the snowball sampling method among the participants. It is important to point out that this population is difficult to access, both in terms of the restrictions that the companies impose on their workers and the resistance on the part of the subjects themselves to openly address sensitive topics, such as their intimate lives, love, and sexuality, which were addressed in each of the interviews. This difficulty was resolved through a process of voluntarily opening up, through conversation, favoring the display of narratives based fundamentally on listening, which opens the possibility for joint reflection with the interviewees.

In this research, we worked under a feminist focus, with a critical perspective, which meant concerning ourselves with seeing that the construction of objectives and questions would open up a field for us to see how power relations are constructed in mining couples within a base dynamic between the subject and society. In the research practice, we worked to include man/woman duplas with academic preparation and a practical background in gender and rights in the different fields of action, in both the production of the interviews as well as the analysis of the interactions and subjective expressions that emerged in the process. The selection of the conceptual framework for the theoretical counterpoint came from the aspect of feminism and critical gender, as can be seen in the list of references.

During the analysis, we identified the inscriptions of the mining culture on the construction of subjectivity and gender, as arrangements achieved in the intimate life and the place of men and women and the tensions due to the sexual division of the work, the control of money, and affective agreements. By understanding these processes, the advances and continuities of mandates in the life of the couple could be analyzed, considering that the viewpoint of the person doing the research associated with the object of study is therefore understood as limited and localized; that is, part of the principle that “our vision is always a question of being able to see” [43].

The confidentiality agreement with the participants was carried out by signing informed consents, in which the objectives and the topics that would be addressed in the interviews were presented. Incentives were not considered for the participants in this group. The entire research process has been submitted for approval by the Ethics Committee of the National Council of Science and Technology of Chile, registered under folio No. 1180079. The criteria for inclusion in the study for women partners of mining workers centered on them being residents of the second region of Chile, with an age range between 18 and 65. For the male miners, that they were from 18 to 65 years of age, heterosexual, with a partner and children, and that they work in mining shift systems.

## 3. Discussion of the Results

**Figure 3:**
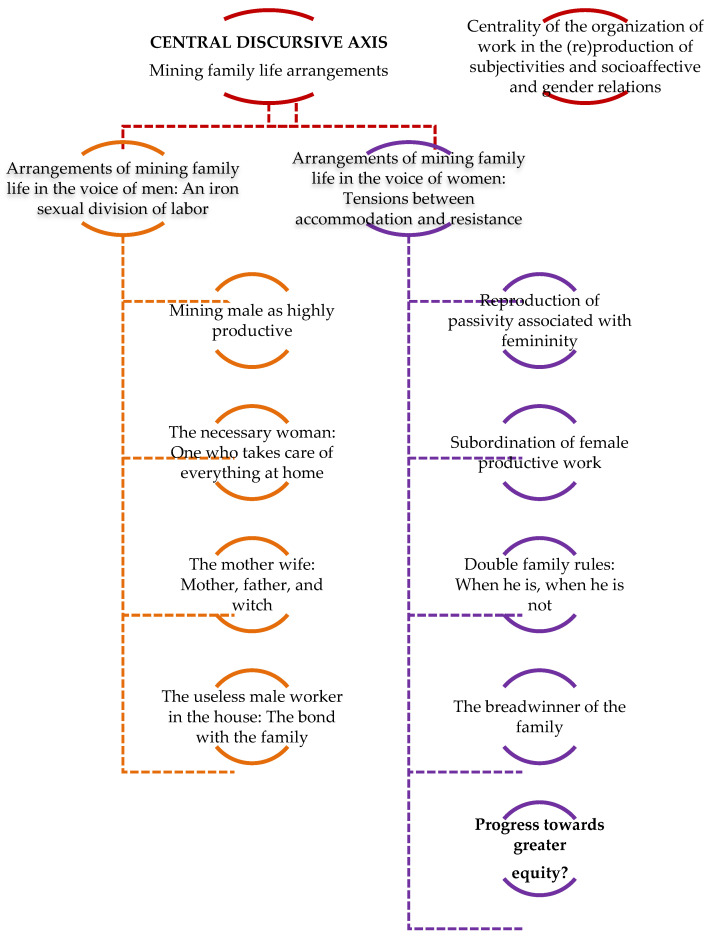
Mining family life arrangements. Source: self-prepared.

We organized the results based on a survey of a broad discursive focus that we denominate “Arrangements of mining family life: The centrality of the organization of the work in the (re)production of subjectivities and socioaffective and gender relations.” With the analysis, we sought to interpret how, through the discourse on work and its organization by shifts, subjective positionings and socioaffective relations are constructed. Based on this central focus, we identified two associated focuses. The first, sustained entirely by the male mining workers interviewed, strictly reproduces a traditional sexual division of the work, practically distributing the production tasks to men and the reproduction tasks to women. The second, confirmed by the statements made by women, gives an account of some tensions, although far from subverting or transforming the gender order that seems to be reproduced with particular strength by the organization of the mining work. We developed a section in which, as we present the interpretations regarding the subjective positions produced by the discourse and link them with fragments of the discourse of the interviewees, we sought to specify the theoretical and epistemological frameworks based on which we made our interpretation proposals. The following is a summary chart that we develop more extensively below (see Figure 3):

### 3.1. Arrangements of Mining Family Life in the Voice of the Men: A Strictly Sexual Division of the Work

In this first section, as we pointed out above, we analyzed fragments of discourse expressed by male miners and we propose interpretations regarding the subjective positions produced.

1. The male miner as highly productive.

We understand the organization of work and gender as sources of demand based on the production of subjectivities and social relations. In this sense, we started from the premise that mining work and its social and technical organization require workers to raise defensive strategies of the trade that are reconfiguring their bodies and subjectivities [26]. Additionally, this subjective configuration often implies an effort of virilization in which the value of productivity holds a central place [27]. The virilization of the subjective body is defined by [42] as a defensive strategy employed by men to protect their masculine identity as well as to endure the psycho-physical demands of the work, which consists of a loss of sensitivity and an emotional hardening on one hand, and a reduction in thinking to a cost–benefit calculation on the other, which enables sustaining the high productivity standards expected by contemporary companies and to endure them as part of the virile virtues [23]. In this regard, we considered the following fragment of an interview:

“You endure it. It’s impossible for a man to solve family matters, but on the job, a mining worker is highly productive! That is, the productivity of a mining worker is very high. We know that data. The men solve problems quickly; it’s technical, you look for ideas, you resolve, investigate. But they are not able to implement those abilities at home.”

(José, 61 years old, three children)

A rhetorical analysis of the extract selected leads us to establish a strong link between two values raised by this interviewee’s discourse. On one hand, the capacity to endure, theoretically defined as the art of not escaping, of bearing whatever comes, a type of sacrifice not exempt of pain that serves to support the image and virile honor [44] and on the other hand, productivity, which, with emphasis on the rhetorical analysis, does not involve just any productivity here, but rather a high productivity. The interviewee also refers to making manifest the knowledge of data that prove that level of productivity as a way of granting veracity to his statement. Productivity and endurance emerge as important meanings in support of the subjectivity of the male miners. However, the same interviewee points out an important controversy: the other side of the miner’s productivity on the worksite is his total unproductiveness in domestic-family tasks. The consequence of that discursive construction of work productivity seems to be the fact that the worksite emerges as an exclusive space for deploying the men’s creativity in search of solutions. We point out that in this extract of the interview, the gender discourse, spoken and being spoken through the interviewee, reproduces the classic sexual division of work and that, in the name of work productivity and endurance on site, the male worker is excluded from the demands of conflict resolution in family life.

2. The necessary woman: one who takes charge of everything at home.

The reproduction of the sexual division of work in the discourse analyzed reappears strongly in the discursive constructions regarding what the men need from their partners:

“The women, in our case, have to take care of everything when we leave. You can’t be going down all the time, asking your boss for permission. You can’t go down, because if you are going to be going down, there are, I don’t know, fifteen of us up there and the boss is counting on the fifteen of us, so if you leave, the other fourteen feel it and have to take on the work of the one who left. So, the idea is to try not to go down, so you don’t look bad with the boss and also to take care of your job. For that reason, our women are important because they have to take care of everything.”

(Mario, 32 years old, one child)

This extract from an interview—selected for being an idea that is repeated in the discourse—reinforces the discursive construction that situates the male worker as being indispensable on the worksite: “You leave and they feel it”, while at the same time he is absolutely replaceable in tasks at home, where “she has to take care of everything.” The construction is polar, practically dichotomic, and reproduces the classical notion of the sexual division of work: The productive dimension of work is the men’s responsibility, while the reproductive dimension of life is the women’s responsibility. This arrangement allows the worker to be completely available for work while on shift, which seems to be fundamental to take care of your job; i.e., to sustain employment and to not look bad to the boss. What management requires is construed as a form of total availability and that is a standard that a worker obtains to the extent that he has a partner who takes care of everything at home. The strict gendered division of work is discursively construed as a type of key requirement for conserving employment (and a counterpart not stated here—family life, of which she is in charge).

As pointed out by [25] in a study with male oil workers, the organization of mining work also produces subjects—in this case a way of experiencing masculinity that converts men into a productive force always available for the worksite. The production system also involves the production of producers [24].

3. The mother spouse as mother, father and witch.

Following the analysis of the discursive construction of the narratives and the positions assigned to men and women in the statements studied, we look at the following interview extracts in order to go into some specifications regarding this “all” referred to in the discourse, especially with regard to the reproductive tasks associated with caring for and raising children:

“The old lady has to know how to substitute at all times (accidents, birthdays, parties, others), when the children are sad the old lady has to deal with the little kids, so they don’t miss us, so while we are on the job, they don’t need anything from us.”

(Pedro, 36 years old, two children)

In general, caretaking tasks, and in this specific case, those that have to do with the affective support of the children, are construed as the woman’s duty, since *the old lady has to*, where “*has to*” expresses an imperative. We say that what is expressed here is an act of speaking, since in saying it, it becomes an imperative. The imperative is manifested as a *deal with the little kids*, which we understand as a requirement to take care of the children’s emotional stability and to fill, on the affective plane, the role of father and mother, since the expected effect of the task assigned to the mother is so the father *is not missed* while he is on the worksite. We could say that the assignment that the interviewee makes here is that his absence is not felt, which constitutes an illusion, or a type of negation of reality. In terms of analysis, what is important for us to point out is again the act of speaking, since it involves a discourse that prescribes the expected tasks of the woman-spouse in terms of affective care of the children.

There is more, however, since this requirement for compliance in tasks of an affective nature also overlaps with those functions that have historically been associated with the paternal role, such as establishing rules and respect for them at home:

“The other thing that happens to me is the matter of the kids. When I come home and they do something bad, I don’t scold them. I don’t feel I have the right to scold them because I am up on the site for seven days. If you come home and scold them, you end up being a bad father figure. So, I leave all that to my wife. My wife does their homework, teaches the children. Everything that has to do with the kids at home, I completely exempt myself (…) They end up being the witches, ha ha ha.”

(Manuel, 40 years old, three children)

Covered and justified by the shift system, the male interviewee allows himself to renounce the tasks of a normative order at home regarding his children, explicitly avoiding them and placing that duty on his partner. In the words of Lagarde [45], they seem to require a mother/spouse who doubles as a *witch*, also in charge of the rules and limits. The discursive recourse to the metaphor of the witch operates as a release and shifting of responsibilities associated with what is ugly and detestable. The witch is put in the place of the one who does the work that the man does not like. In this case, the educational and normative work regarding the children. However, the inverse of this discursive recourse is that the male mining worker is losing his place at home. We propose that the organization of mining work in the greater northern region of Chile produces producers who are indispensable on the worksite and totally dispensable in family life.

Another important matter to clarify is that this arrangement is constructed by the man in solitude, or at least constitutes an agreement not necessarily spoken. He does not tell her “You be the witch”; he does it, up to a certain point behind her back, and that seems to be expressed by the laugh: “*They end up being the witches, ha ha ha,*” a *they* that is also pronounced in an impersonal matter, since it does not involve an agreement made with the other. It is a tacit agreement and perhaps taken as obvious, as a requirement and “natural” consequence of the man’s work system, which reproduces gender order based on male domination and its symbolic violence [23].

4. The male worker who is useless at home: money as a link to the family.

We propose that the discourse configures the following image: We need a woman/mother/spouse who does not need us; with that, the man constructs himself as a subject absolutely dispensable at home, and even undesired:

“After the shift, you come home to a house that your wife dominates. Your children don’t pay much attention to you. If it is you that tries to impose something, in the end, the only thing they want is for you to go back up to the mine. You become a provider, a provider and nothing, nothing else.”

(Claudio, 38 years old, three children)

“I come back and the youngest pretends she doesn’t know me.”

(Patricio, 42 years old, two children)

A circle seems to be closing in which the discourse reproduced by the interviewees is the strictest gendered division of work. However, a very important detail is that this exercise does not allow them to be positioned at home as a figure of power. On the contrary, the subjective sensation grates on contempt and rejection, on not wanting him to be there, for him to go back to the worksite, to the place where he is truly needed and important, and where his subjectivity makes sense and can be sustained. At home, the *wife dominates,* and the children do not recognize the father’s authority. Constructed that way, the negotiation between the members of the couple ends up excluding the male worker from the intimacy of family life, constructs him as a provider and a type of stranger. Money and his contributions as a provider constitute the measure of the bond between the male worker and his home and family.

### 3.2. Arrangements of Mining Family Life in the Voice of the Women: Tensions between Accommodation and Resistance

This focus principally surveys the discourse of the female partners of mining workers. It is also centered on the discursive construction of the gendered division of work and the issues relative to economic and affective dependence (or not) regarding the father—provider figure.

Just as in other Latin American cultures [45], this hierarchical and gendered order within the couple is naturalized in the mining culture, granting and reinforcing the sense of the forms of a heteropatriarchal nuclear family construction in the zone. Additionally, the subjective positionings constructed for women in the private sphere of the home and at a considerable distance from the places of decision-making and productive work are actualized [46,47,48,49].

5. Reproduction of passiveness associated with females.

We looked at this first fragment, in which it appears that the strategy constructed to carry on life as a couple seems to be that of more or less passive accommodation to the sexual division of work proposed (or imposed) by the shift system:

“When he is at home, we don’t get bored because we say, “What are we going to do today?” “Let´s go for a walk,” we have to buy something. I don´t like to go downtown by myself unless I have to do an errand, and I don´t like to be in a line but I have to, and I do it with my head down. I don’t like to go window shopping either or have a coffee. If I have to visit my sister, my friend, or sister-in-law, I dare to go. He takes me everywhere. If I say, “I don´t want to cook,” he tells me, “Let’s eat out.”

(Mónica, 43 years old, two children)

The participant presents a couple relationship in which she remains in a place of waiting and passivity and he, in a position of action and even diversion. The text is explicit: “When he is at home we don’t get bored”. What is left unsaid, to say it that way, the inverse of this grammatical expression is that when he is not there, she does get bored, since going out alone into the public space (doing errands, window shopping in the mall, having a coffee) seem to be a prohibited act, one that you must do with your head down, so as not to be seen. This fragment, selected, once again, because it is repeated in the discourse, constructs and reproduces the public space as a space prohibited for a woman who is not accompanied by a man. For reasons deeply rooted in gender order and male domination [23], the woman only goes out alone if she gets together with other women, but in private and family spaces (with a sister, sister-in-law, or at most, with a friend). The discourse reproduces subjective positionings for women that are based on relations of emotional and economic dependence toward the male provider figure, actualizing feelings of insecurity and fear when faced with occupying a public space. The man is construed as a protector and dispenser of monetary resources for consumption that provide satisfaction [50]. This discourse supports the restriction of women’s possibilities for developing life plans independent of the couple [46]. It discursively reproduces a model of a couple sustained by the image of romantic love through which an ideal of completeness is promoted and normalized based on a hierarchized and stereotyped dichotomy of (supposedly) male and female roles [49,51]. A type of distribution of space that reproduces restricted modes of subjectivation in the symbolic-material space of the couple: dependence, fear, insecurity and infantilization of the woman, and in the man, control and management of the couple’s life. It is the organization of mining work and its production, especially based on a shift system, that ends up also organizing the life of the woman interviewed, since her day-to-day life is structured based on the presence/absence of her partner at home and in the city.

6. Subordination of female productive work.

The discourse analyzed is not necessarily monolithic. In this regard, we reviewed the following extract, which seems to focus on the women’s possibilities of performing productive tasks as a focus of transformation and gain in gender equality:

“Since last year, I own a SME (small-to-medium enterprise) related to pastry. At the beginning, it was difficult because I was taking his time (from him when he was at home). I noticed, along with a group of girls, that when they come down from the mine, you have to be completely available for them because they have been confined. That’s what they think. When they come down, they like to go out. My husband likes spending time with his family. Last Friday, I had to deliver 400 sweets. I had told him that I wanted be at home early to start preparing my sweets in peace. He made us go out early, but I got back at 8 PM. I felt cheated because we did what he wanted to do anyway. So, it’s here where you have to give in a lot, so you don’t quarrel.”

(Silvia, 35 years old, three children)

In this case, difficulties are observed in establishing negotiations with the partner that enable delimiting individual and work development spaces in favor of the woman. The fragment chosen presents a power relationship in which she has to make significant efforts to accommodate him, constructing the image of their couple in the mode that they (the group of mining couples, *with a group of girls*) seem to understand it—as men who demand the availability of (their) women when they are at home. The foregoing seems to assume a willingness to suspend any other interest or task, which places them in a position of subordination with respect to the male worker’s needs at home. The woman is placed in a position where she has to give in to the demands for time and attention required by her partner and the manifested objective for that is to avoid conflict. For the participant, assenting to her partner’s requirements implies postponing both her personal interests and her search for economic and affective independence [45,47,52]. In this way, a subordination is reproduced toward the symbolic and economic control exercised by the provider figure. The quote is explicit: Despite her request to consider her needs too (return early), she is not heard. The way the phrases are constructed is also eloquent: *He made us go out early*, *we did what he wanted to do anyway*. The action and the decision-making capacity continue being that of the man’s, and the woman, at least in this interview fragment, accommodates those requirements in order to avoid a fight. In this case, the woman’s economic entrepreneurship is possible, but provided it is subordinated to the role of mother/spouse and is clearly hindered when the man is at home. We would say that the work organization that the women gives to her small pastry business is traversed by the shift system that organizes the time of her husband, a mine worker. Her main role, when her husband is home, is discursively constructed as making herself available for the husbands’ requirements.

It is important to state that, while tension is manifested, the possibility of making an issue of it or fighting to transform the situation is not discerned in the discourse. The manifest is imperative; that is, what she *has* to do is *to give so as not to quarrel*, and so as not to manifest dislike, it is probably to safeguard the worker’s physical and psychological rest, since he will soon return to the worksite.

7. Dual family rules: when he is at home and when he is not.

Finally, another nuance of the discourse is what we found in the following fragment, in which we can appreciate a type of acceptance of different rules, one with the husband and father at home and another without him:

“When he (husband) is at home, everything changes. For instance […], he doesn´t eat vegetables so I have to cook pasta. When my son and I are alone, I cook other types of meals. […] I don’t make plans when he´s at home. A woman comes to the house to iron when he is not at home because he doesn´t like strangers at home. When he is not at home, I make the rules, but when he arrives home, everything changes. For instance, I don’t leave things on the dinner table, but he leaves his shoes anyplace, his bag at the door until he is leaving, and he just unpacks it that day. I used to unpack it for him and put the things away, but not anymore. It´s another rhythm when he´s here, it´s something different at home.”

(Estela, 50 years old, three children)

Just as in the previous case, we can propose that this involves tacit agreements, that the woman accepts in silence, as a type of passive resistance rather than the mode of an exercise of adaptation; she does not dispute it for its form, she exercises it when the man is not there. The fragment is eloquent: “I don’t make plans when he’s at home”. We interpret this as a type of need for subjective suspension by the woman: there are no plans, there is no possibility of deciding on something when he is at home. It would seem that all rights and possibilities for acting are his. The interviewee speaks of two different ways of living at home that are marked by the absence/presence of the male-father-worker at home. We could say two different cultures, two different sets of rules, that the interviewee refers to, from orderliness to what they eat. We propose that the discourse speaks of the subjective positioning of the woman in the family culture, stressed between obedience to the demands and requirements of her partner and seeking autonomy in the relationship and in her home. The absence of the male figure is construed as the chance to organize the family space according to her priorities, while his presence is practically a time to be endured: he leaves his shoes anyplace, his bag at the door until he is leaving. Once again, the organization of mining work is also the organization of affective and family life. It is the guideline for what the woman can and cannot do at home at different times of the shift.

What we previously denominated “mining family normativity” constitutes a social ordering through which gender order is reproduced. We propose that this normativity forms part of what Meler [53] called the “mining family culture”, according to which the constant adjustments experienced in the intimate life of the couple and in their relationship with their children derived from the shift work and the organization of mining work in general are subjectivized, i.e., incorporated into the psyches and bodies of the family members, thereby defining their ways of being and relating in the world [53]. As a corollary, we posit that the social and technical organization of mining work reproduces the heteropatriarchal gender order, or rather, that the traditional gender order is reproduced by the mining organization of work.

8. The mother as the support for the family.

As proposed by Dejours and Gernett [54], as well as Meler [53], the organization of work also reaches the processes of subjectivation of the children, since it traverses the patterns of child-raising and defines the styles of bonds that impact the production of the children’s subjectivity. For that, we considered the following interview fragment:

“I am the witch, and he (the husband) is the boy who plays with him (the son) all day. Even last year, Vicente (son) was called on (at school) to recognize the family: I am the mother, and he is the father and the brother. The other day I answered him (her partner) and told him, “Thanks to me he loves you, because since he was a baby, he didn’t catch you [didn’t take him into account].” The child must have felt that he rejected him, since he wasn’t affectionate. They weren’t like they are now, that they are more like partners, more united. If one doesn’t feel the love from the other person, he is indifferent. That’s the feeling in their relationship. Over time, I insisted so much with him on Vicente that now they get along better. Now Vicente is bigger; I taught him to play ball, with cars—it wasn’t him.”

(María, 45 years old, one child)

The signifier *the witch* (re)appears, this time in the voice of a woman interviewee. Here, the witch represents the person who has to take charge of everything except playing with the child, which, as a counterpart, emerges here as the exclusive task of the father. The discourse emphasizes that by bringing an image that speaks of an exercise that the child does at school, in which he recognizes his father also as his brother, alluding to the place of parity in which the male worker is positioned with respect to his son. In this way, the discourse situates the woman in a position of authority, more of an uncomfortable authority that is signified with the word *witch*.

Moreover, the narrator positions herself as a guarantor of the love between father and son. It is she who articulates it, who promotes it, and further, thanks to her, the child loves the father. The man is signified as somehow incapable of generating love or attachment with the child, like he is unable, even though, now, fathers are not like they were before: *They are more like partners, more united.* Despite these cultural changes that the interviewee recognizes in the world, they involve changes that her husband, male mining worker, has not been able to develop in himself. If there are advances in this transformation, they are the result of her efforts. This fragment takes us back to the first text chosen, in which the male mining worker is creative and productive on the worksite, but incapable of contributing at home. The workforce, the productive force remains in the company’s absolute possession.

9. Advances toward greater equality?

Finally, as part of the women’s discourse, we find experiences and subjective positionings that somehow tend toward equality; however, this trend does not seem to be sustainable. The extract that we review below is from a 32-year-old women who worked in mining but stopped because of what she construes as a sort of natural agreement in the organization of family and affective life:

“We both worked 4 × 3, and I was practically in charge of taking care of things at home regarding the bills and tasks that take a little longer (…). Regarding the house, we both took care of day-to-day or domestic things equally. And now that he is 4 × 3 and I am in Antofagasta, we turned things around. He is in charge of tasks that take a little longer, which are done on Fridays, and I am in charge of the house and the more urgent things that have to be done in Antofagasta (…). He generally makes the investment decisions or I consult with him so he knows, but that happens naturally. The same goes for family matters. I am the one in charge of the house, what to buy, what we need, what has to be put and what has to be taken out. It happens naturally (…) and we agree that way and get along well.”

(Marcela, 32 years old, one son)

The advance toward gender equality in this fragment can be found at the time when they both worked in mining. In that context, the discourse presents a certain contradiction, since on one hand it points out a sort of parity in the distribution of tasks at home, although at the same time she is explicit in stating “I was in charge of taking care of things at home…” What is not manifested in the narrative is what motivated the women to leave work. In this respect, the research of [27], in a study performed with general managers of companies, gives account of how, in work backgrounds that are comparable between men and women in a couple, the birth of a child is key to generating an imbalance, since in a supposedly natural manner, as also expressed in the fragment in question, it is the woman who is in charge of child care.

In any case, an effort of female subjective positioning can be seen in this fragment, in a framework of greater equality, or at least of a manifest exercise of construction of agreements based on negotiation instead of a passive acceptance of the conditions. However, two things should be pointed out. The first is regarding the use of money, but not just of any money. It involves money that Clara Coria [55] in her study on the hidden sex of money denominates “big money” or that destined for investments, and not that used in the day-to-day administration of the home, which is defined as “small money”. In this case, these decisions continue to be made according to the classical division proposed by the author: big money is the mens’ affair, while small money is the women’s. The second, which we insinuated above, is that the discursive construction of the family order is signified by the interviewee as “natural”: “it happens naturally” that she takes charge of day-to-day things of the home. We propose that the discourse reproduced here is that of gender on its more traditional axis, naturalizing the positioning of women in domestic and reproductive tasks, and men in public and productive work. The shift system and the social and technical organization of mining work continue to not be seen as agents that produce subjectivities and social and family relations.

## 4. Conclusions

Our research question was: Which cultural impositions of a gender nature are reproduced, and which are transformed in the discourse of mining couples?

In this regard, we would say that the so-called mining family culture; that is, the ways of intimate life, of organizing family relations and even the possibilities of experiencing oneself, of feeling one’s own subjectivity, in the frameworks that the organization of mining work, and especially the shift system proposes or imposes, favor the reproduction of a gender order strongly marked by male domination based on a strict sexual division of the work, that assigns production tasks to the men and reproduction to the women, placing the former in a position of greater value and around whom family life is organized.

We also discovered that the majority of the arrangements described in the discourse do not, strictly speaking, constitute agreements, since most of the time they are tacit, not negotiated, and perhaps taken as obvious or natural. The possibility for arrangement is paired with fear of conflict, especially in the women, who seem to be forced to maintain an emotional climate as free of tension as possible at home, perhaps as a way of guaranteeing optimal rest for the male worker, thereby benefitting his optimal return to the productive tasks that provide profitability for the company.

For the male workers, the effort to endure this work organization seems to involve a significant risk, which is the sense, often explicit in their work, that the quality of life of their family ends up being the cost of their work. They work for the family at the cost of family life. The interviewees reiterate and reproduce a type of paradox: needing a family that does not need them, which ends up positioning the men in the only place possible—the worksite. It is there where they feel they are contributing, capable of offering solutions, of mobilizing their intelligence and their senses in the interest of resolving the problems that the daily work presents, which is often not the case at home and in issues of a domestic nature, since there it is she who must deal with it.

We will also state that since this involves a more exploratory, situated and qualitative research that seeks to delve into matters of the production of subjectivities, it does not intend to generalize its conclusions, but, to take them seriously if we wish to advance in labor policies that benefit the overall health of the workers on one hand, and the advance of gender equality on the other.

Finally, we started this research with the idea of finding tensions and escape points, cracks or fissures in the standard gender order in the contemporary, post-feminist revolution mining family in Chile. Perhaps we expected to find more crucial discursive positions, especially among women and in younger couples. However, in the demands of the organization of mining work in Antofagasta, we found that there is still a type of quarry, reproducing male domination—a matter that, in symbolic terms, constitutes a significant challenge for the country, considering that mining is the principal local economic activity.

## Figures and Tables

**Table 1:** Example matrix of narrative analysis.

Dimension: Referred to the Central Topic of Analysis						
Knots and conflicts	Subjects	Narrative	Subjective and gender experience tensions	Linked emotions	Search objects	Synthesis of interpretative analysis in the theoretical counterpoint
Identification of knots and their ramifications; coding of the interview; sex of the participant; age; microtexts and school assignment to selected text published extensively.	Coding of the interview; sex of the participant; age; attachment to school.	Microtexts and selected texts published extensively.	Analysis of the major emerging conflicts; microprints of the research objectives; articulated analysis of the theoretical core of the research.	Sensations and feelings that emerge in the affective–emotional story of the subjects.	Subjective expressions of the participants from a situated position.	Analysis of the theoretical core of research; counterpoint three levels: The protagonist, the researcher, and the theoretical aspects.
Synthesis of the analysis of the dimension in three voices: interviewee theories, and researcher.						

Source: Self-prepared.

**Table 2:** Participants in the study.

	Code	Age	Occupation	Shift System	Socioeconomic Level	No. of Children
**1**	José	61	Operator—union	4 × 3	Medium–low	3
**2**	Pedro	36	Mining worker	7 × 7	Medium–low	2
**3**	Claudio	38	Mining worker	7 × 7	Medium–low	3
**4**	Patricio	42	Mining worker	4 × 3	Medium–low	2
**5**	Manuel	40	Operator—union	4 × 3	Medium–low	3
**6**	Mario	32	Mining worker	4 × 3	Medium–low	1
**7**	Mónica	43	Homemaker	-	Medium–low	2
**8**	Silvia	35	Small business owner	-	Medium–low	3
**9**	Estela	50	Homemaker	-	Medium–low	3
**10**	María	45	Homemaker	-	Medium–low	1
**11**	Marcela	32	Homemaker (worked in mining)	.	Medium–low	1

Source: self-prepared.

## Data Availability

The data supporting the findings of this study are available on request from the corresponding author (J.S., FONDECYT 1180079). The data are not publicly available due to their containing information that could compromise the privacy of research participants.

## References

[1] Observatorio Laboral Antofagasta (OLAB) (2017). Reporte Laboral Sectorial.

[2] Instituto Nacional de Estadísticas Chile (INE). https://www.ine.cl/prensa/2019/09/16/antofagasta-magallanes-y-o-higgins-lideraron-el-crecimiento-econ%C3%B3mico-interanual-en-el-primer-trimestre-de-2018.

[3] Ivanova G., Rolfe J., Williams G. (2011). Assessing development options in mining communities using stated preference techniques. Resour. Policy.

[4] Rivera N., Aroca P. (2014). Escalas de Producción en Economías Mineras: El caso de Chile en su Dimensión Regional. Observatorio Regional de Desarrollo Humano. La Encuesta CASEN 2015: Algunos Resultados de Interés Para la Región de Antofagasta. EURE Santiago.

[5] Silva-Segovia J., Salinas-Meruane P. (2016). With the mine in the veins: Emotional adjustments in female partners of Chilean mining workers. Gender Place Cult..

[6] Hernández G., Pávez J., Rebolledo L., Valdés X. (2014). Trabajos y Familias en el Neoliberalismo: Hombres y Mujeres en Faenas de la UVA, el Salmón y el Cobre.

[7] Urrutia V.G., Figueroa A.J. (2015). Corresponsabilidad familiar y el equilibrio trabajo-familia: Medios para mejorar la equidad de género. Polis Santiago.

[8] Angelcos N., Pérez M. (2017). De la “Desaparición” a la reemergencia: Continuidades y rupturas del movimiento de pobladores en Chile. Latin Am. Res. Rev..

[9] Ruiz S., Grande M.L. (2015). Participación política y liderazgo de género: Las presidentas latinoamericanas. América Latina Hoy..

[10] Caro P., Román H., Armijo L. (2020). Cuerpos de mujeres, significados de género y límites simbólicos en la gran minería en Chile. Polis.

[11] Silva J., Barrientos J. (2008). Guiones sexuales de la seducción, el erotismo y los encuentros sexuales en el norte de Chile. Rev. Estud. Fem..

[12] Pini B., Mayes R. (2012). Gender, emotions and fly-in fly-out work. Aust. J. Soc. Issues.

[13] Winn P., Klubock T.M. (1999). Contested communities: Class, gender, and politics in Chile’s El Teniente copper mine, 1904–1951. Am. Hist. Rev..

[14] Salinas P., Reyes C., Romani G., Ziede M. (2010). Mercado laboral femenino. Un estudio empírico, desde la perspectiva de la de-manda, en la región minera de Antofagasta. Innovar.

[15] Silva-Segovia J., Lay-Lisboa S. (2017). The power of money in gender relations from a Chilean mining culture. Affilia.

[16] Montecino S., Rebolledo L., Sunkel G. (1999). Análisis del Impacto Psicosocial de los Sistemas de Trabajo por Turnos en la Unidad Familiar.

[17] Pini B., Mayes R., Boyer K. (2013). “Scary” heterosexualities in a rural Australian mining town. J. Rural. Stud..

[18] Hubbard P. (1999). Researching female sex work: Reflections on geographical exclusion, critical methodologies and ’useful’ knowledge. Area.

[19] Hubbard P., Sanders T. (2003). Making space for sex work: Female street prostitution and the production of urban space. Int. J. Urban Reg. Res..

[20] Hubbard P. (2004). Revenge and injustice in the neoliberal city: Uncovering masculinist agendas. Antipode.

[21] Carrasco C., Vega P. (2011). Cuaderno de Investigación N° 40: Una Aproximación a las Condiciones de Trabajo en la Gran Minería de Altura.

[22] Bourdieu P. (2000). La Dominación Masculina.

[23] Olavarría J., Olavarría J. (2009). Globalización, Género y Masculinidades. Las Corporaciones Transnacionales y la Producción de Productores. En Masculinidades y Globalización: Trabajo Y Vida Privada, Familias Y Sexualidades.

[24] Palermo H. (2017). La Producción de la Masculinidad en el Trabajo Petrolero.

[25] Dejours C. (2012). Trabajo Vivo. Tomo 1. Sexualidad y Trabajo.

[26] Pastor P.Z., Palermo H., Capogrossi M.L. (2020). Reproducción de la dominación masculina en la subjetivación del trabajo. Un análisis de discurso de gerentes generales en el Chile anterior a la explosión social. Tratado Latinoamericano de Antropología del Trabajo.

[27] Leiva S., Comelin A. (2015). Conciliación entre la vida familiar y laboral: Evaluación del programa IGUALA en una empresa minera en la región de Tarapacá. Rev. Polis.

[28] Connell R., Masserschmidt J. (2005). Hegemonic masculinity: Rethinking the concept. Gend. Soc..

[29] Gilmore D. (1994). Hacerse Hombre. Concepciones Culturales Sobre Masculinidad.

[30] Zerán F. (2018). Mayo Feminista. La Rebelión Contra el Patriarcado.

[31] Richards N., Zerán F. (2018). La insurgencia feminista de mayo 2018. Mayo Feminista. La Rebelión Contra el Patriarcado.

[32] Denzin N.K. (2012). Triangulation 2.0. J. Mix. Methods Res..

[33] Canales M. (2014). Escucha de la Escucha. Análisis e Interpretación en la Investigación Cualitativa.

[34] Silva J. (2012). La Circulación del Poder Entre Mujeres Chilenas de dos Generaciones: Las Hijas y Las Madres.

[35] Silva J. (2019). Cuerpos Emergentes: Modelo Metodológico Para el Trabajo Corporal Con Mujeres.

[36] De Villers G. (1999). La historia de vida como método clínico. Proposiciones.

[37] Musitano J., Surghi C. (2020). Dossier: La dimensión biográfica. LA Palabra.

[38] Van Dijk T., Wodak R., Meyer M. (2003). La multidisciplinariedad del análisis crítico del discurso: Un alegato a favor de la diversidad. Métodos de Análisis Crítico del Discurso.

[39] Montero A., Canales M. (2013). El análisis francés del discurso y el abordaje de las voces ajenas: Interdiscurso, polifonía, heterogeneidad y topos. Escucha de la Escucha. Análisis e Interpretación en la Investigación Cualitativa.

[40] Davies B., Harré R. (2007). Posicionamiento: La produccion discursiva de la identidad. Athenea Digital Revista de Pensamiento e Investigación Social.

[41] Pastor P.Z. (2018). Reproducción de la Dominación Masculina en la Subjetivación del Trabajo. Ph.D. Thesis.

[42] Haraway D. (1995). Ciencia, Cyborgs y Mujeres. La Reinvención de la Naturaleza.

[43] Abarca H., Sepúlveda M., Ferrándiz F., Feixa C. (2005). Barras bravas. Pasión guerrera. Territorio, masculinidad y violencia en el fútbol chileno. Jóvenes Sin Tregua: Culturas y Políticas de la Violencia.

[44] Lagarde M. (2000). Claves Feministas Para la Autoestima de las Mujeres.

[45] Llombart M.P., Leache P.A. (2010). The gender binarism as a social, corporal and subjective “dispositif” of power. Quad. Psicol..

[46] Fraisse G. (2003). Los dos Gobiernos: La Familia y la Ciudad.

[47] Pisano M. (2001). El Triunfo de la Masculinidad.

[48] Valcárcel A. (1997). La Política de las Mujeres.

[49] Illouz E. (2007). Intimidades Congeladas. Las Emociones en el Capitalismo.

[50] Giddens A. (1992). The Transformation of Intimacy. Sexuality, Love & Eroticism in Modern Societies.

[51] Giddens A. (1998). Sociología.

[52] Lagarde M., Maquieira V. (2010). El derecho humano de las mujeres a una vida libre de violencia. Mujeres, Globalización y Derechos Humanos.

[53] Meler I. (2004). Género, trabajo y familia: Varones trabajando. Subj. Procesos Cogn..

[54] Dejours C., Gernett I. (2014). Psicopatología del Trabajo.

[55] Coria C. (1986). El Sexo Oculto del Dinero. Formas de la Dependencia Femenina.

